# An 18-Year Dataset on the Clinical Incidence and MICs to Antibiotics of *Achromobacter* spp. (Labeled Biochemically or by MAL-DI-TOF MS as *A. xylosoxidans*), Largely in Patient Groups Other than Those with CF

**DOI:** 10.3390/antibiotics11030311

**Published:** 2022-02-25

**Authors:** Claudio Neidhöfer, Christina Berens, Marijo Parčina

**Affiliations:** Institute of Medical Microbiology, Immunology and Parasitology, University Hospital Bonn, University of Bonn, 53127 Bonn, Germany; christina.berens@ukbonn.de (C.B.); parcina@uni-bonn.de (M.P.)

**Keywords:** *Achromobacter xylosoxidans*, *Achromobacter*, *Alcaligenaceae*, *Burkholderiales*, non-fermenting Gram-negative bacilli, emerging pathogens, opportunistic pathogens, nosocomial pathogens, antibiotic resistance

## Abstract

*Achromobacter* spp. are intrinsically multidrug-resistant environmental microorganisms which are known to cause opportunistic, nosocomial, and sometimes chronic infections. The existing literature yields scarcely any larger datasets, especially with regard to the incidence in patient groups other than those with cystic fibrosis. The aim of this study was to fill this gap. We present a retrospective analysis of 314 clinical and 130 screening isolates detected in our diagnostic unit between 2004 and 2021, combined with patients’ demographic and clinical information (ward type and length of hospitalization), and the results of routine diagnostic antibiotic MIC determination. We found the apparent increase in prevalence in our diagnostic unit, in which cystic fibrosis patients are an underrepresented group, in large part to be attributable to an overall increase in the number of samples and, more importantly, changes in the diagnostic setting, such as the introduction of rigorous screening for Gram-negative multidrug-resistant pathogens. We found these *Achromobacter* spp. to be most commonly detected in urine, stool, wounds and airway samples, and found the resistance rates to vary strongly between different sample types. Intestinal carriage is frequently not investigated, and its frequency is likely underestimated. Isolates resistant to meropenem can hardly be treated.

## 1. Introduction

*Achromobacter* spp. are non-fermenting, oxidase- and catalase-positive, motile Gram-negative bacilli that are widespread in nature, especially in moist soils and water sources but also in plants. From a human health perspective, *A. xylosoxidans* especially is considered an emerging nosocomial pathogen capable of causing a wide range of different human infections and morbidities [1]. These typically occur in cystic fibrosis patients [2,3] but are not limited to these, and can range from urinary tract and eye infections to infections of the endocardium, mediastinum, lower respiratory tract, and even the central nervous system and the bloodstream [4,5,6,7,8,9,10,11]. This bacterial species can survive in adverse environments such as ultrasound gels, dialysis fluids, and hospital surfaces and equipment, predisposing it to emerge as an important nosocomial pathogen. As such, it might be considered a potential threat, especially to immunocompromised patients [12,13,14]. The prevalence rates of *Achromobacter* spp. isolates from respiratory samples of cystic fibrosis patients have been reported as increasing in recent years, which has been attributed to selection pressure from antibiotic therapy and the longer survival of cystic fibrosis patients [15]. All of the evidence highlights a growing need for clinical data, as well as in depth analyses on the potential pathogenicity and antibiotic resistance profile of this emerging pathogen [1,15,16]. We contribute to the matter by providing a 444-isolate dataset (314 clinical and 130 screening isolates) in which *Achromobacter* spp. were isolated at our microbiological diagnostic unit at the University Hospital in Bonn between January 2004 and December 2021, and was characterized as *A. xylosoxidans* by MALDI-TOF MS and/or biochemically. Given that only MLST-typing or nrdA gene sequencing allow the accurate species identification of isolates in the genus *Achromobacter*, the isolates will be referred to throughout the text as *Achromobacter* isolates. We complement the dataset with an in-depth analysis thereof, with the aim of further elucidating the antibiotic resistance profile and clinical incidence of *Achromobacter* spp. involved in human infections.

## 2. Results

### 2.1. Specimens in Which Achromobacter Isolates Were Detected 

**Figure 1:**
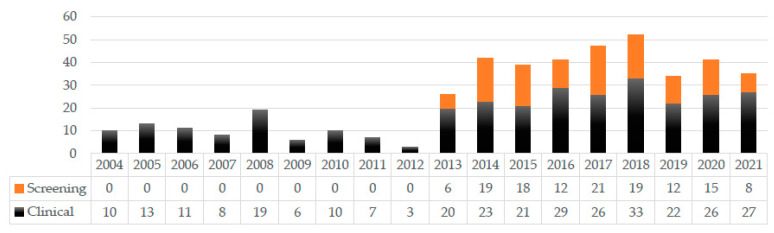
Number of *Achromobacter* isolates detected in the clinical and screening specimens each year.

Between 1 January 2004 and 6 December 2021, *Achromobacter* isolates were detected in 314 clinical and 130 screening samples. Figure 1 depicts the number of isolates detected each year. Starting in 2013, screening for Gram-negative multidrug-resistant pathogens was markedly expanded; as highlighted in the figure, several *Achromobacter* isolates were subsequently unintentionally detected in screening specimens, as they are intrinsically resistant to the cefpodoxime contained in the selective media used, and any species growing on the selective media is routinely identified and resistance tested. There were no *Achromobacter* outbreaks in the mentioned period; hence, the clonality of the isolates was not investigated. The isolates were detected in descending order in the following clinical samples: urine samples (69), stool samples (66), wound samples (57), tracheal secretions (48), nasopharyngeal swabs (48), inguinal swabs (39), anal swabs (29), sputum samples (18), skin swabs (eight), contact lenses (13), ear canal swabs (11), bronchio-alveolar lavages (nine), blood cultures (six), conjunctival swabs (three), tissue samples (two), ascites fluid (two), cerebrospinal fluid (one), vitreous body fluid (one), punctate fluid (one), a continuous venous catheter sample (one), a continuous ambulatory peritoneal dialysis fluid specimen (one), a urethral swab (one), and a sonication (one). Nine isolates were not attributable to patients but to clinical cell culture supernatants.

Given that the deduction of the clinical relevance of a finding from the specimen type alone can be misleading, we included data on the microbiological assignment received by the laboratory upon sample submission in order to further reduce the risk of misinterpretations.

**Figure 2:**
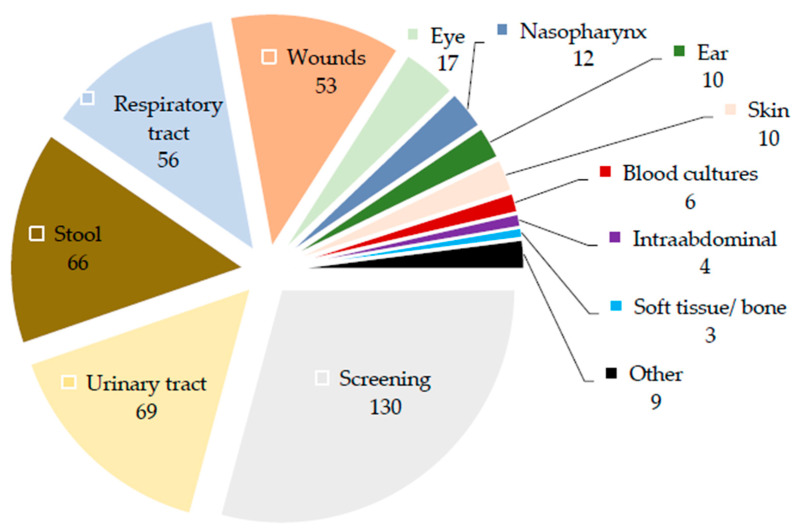
Number of *Achromobacter* isolates detected in the different specimen types.

According to this, *Achromobacter* isolates were detected in 133 screening samples (three clinical samples had “screening” as assignment); 75 respiratory tract samples, of which at least 48 were from intubated or tracheotomized patients; and 69 urinary tract samples. Of these 69, all but a few came with the diagnostic mission of pathogen and susceptibility determination; in at least 15 samples, the *Achromobacter* isolates were the only detected microorganisms. Another 51 isolates were detected in wound samples that—although not necessarily indicating a wound infection—indicated the presence of this organism in the actual wound. In at least 15 wound samples, *Achromobacter* isolates were the only detected microorganisms. Fifteen samples indicated a potential eye infection, six samples indicated bacteremia, five samples indicated an intraabdominal infection, three samples indicated a soft tissue/ bone infection, 10 samples indicated an infection of the ear, and one sample indicated a central nervous system infection. The amount of *Achromobacter* isolates detected in the different specimens is displayed in Figure 2. Among the screening specimens, the most frequent were inguinal swabs (37), followed by pharyngeal swabs (30), anal swabs (29), and tracheal secretions (19).

### 2.2. Minimum Inhibitory Concentrations

**Figure 3:**
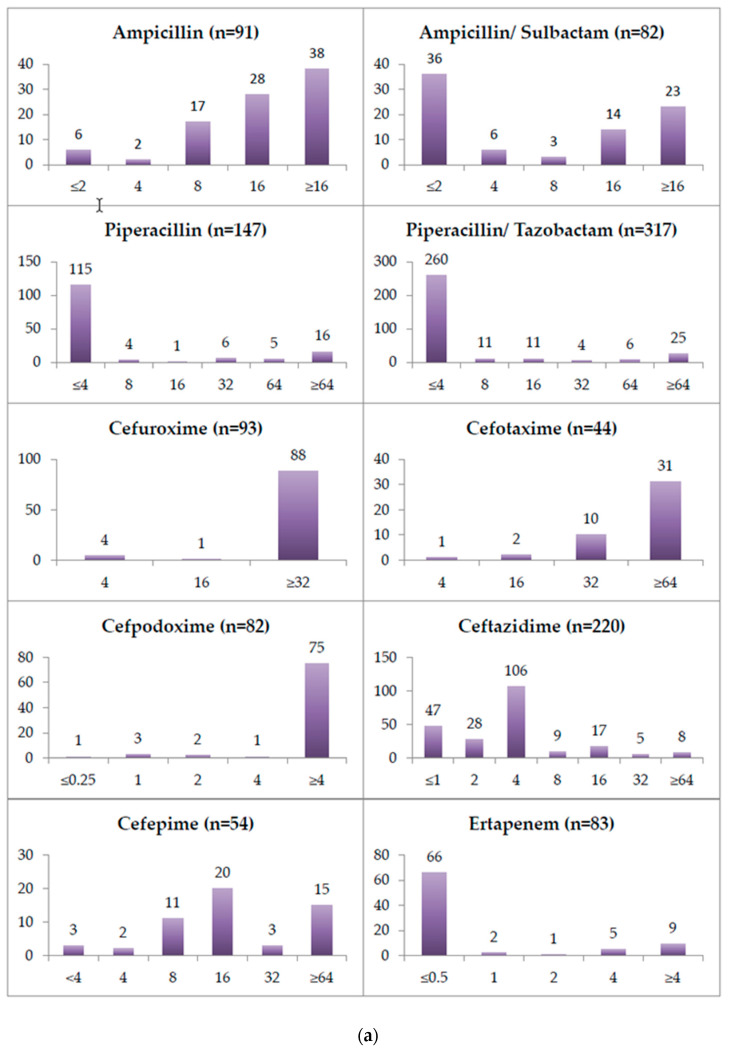

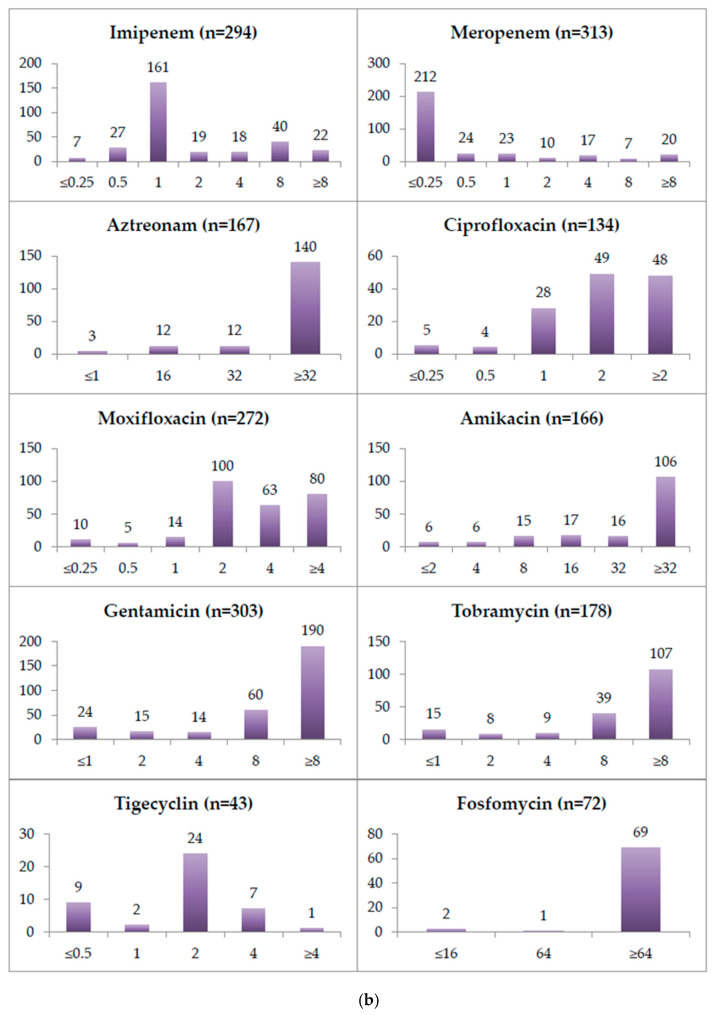

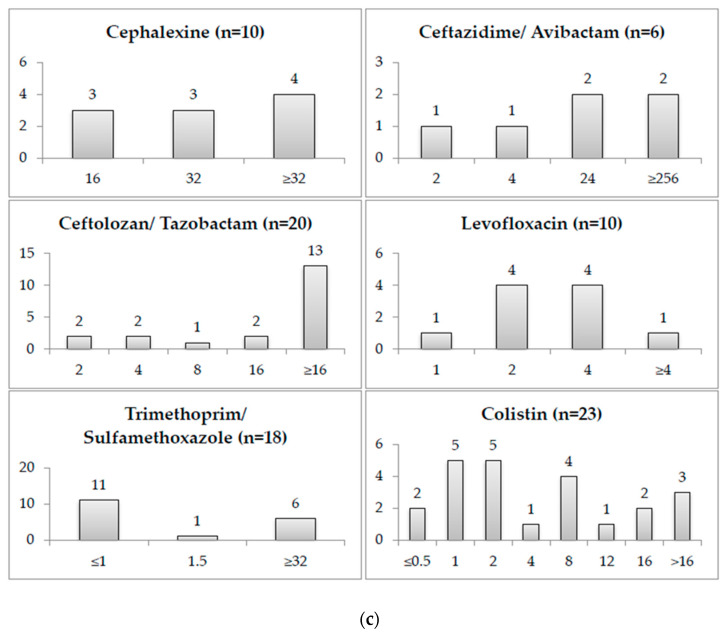
(**a**) Minimum inhibitory concentrations (MICs) for the tested antibiotics (part 1). (**b**) Minimum inhibitory concentrations (MICs) for the tested antibiotics (part 2). (**c**) Minimum inhibitory concentrations (MICs) for the tested antibiotics (part 3). The gray bars indicate a low sample size.

The minimum inhibitory concentration (MIC) for all of the tested antibiotics for the first isolates tested between January 2004 and December 2021 is depicted in Figure 3. These were 333 isolates. Starting in 2013, antibiotic susceptibility testing in the laboratory was routinely performed by means of the VITEK 2 system (biomérieux, Marcy-l’Etoile, France). The great majority of all of the *Achromobacter* isolates from 2004 until 2012 were tested by disc diffusion, which precludes MIC determination. This limits the data on MICs in the period of 2004 to 2012 to those exceptional cases in which the testing was performed using gradient-strip tests. All of the MIC values for colistin included in the study were determined by microdilution. According to EUCAST Clinical Breakpoint Tables v. 11.0 (valid from 2021-01-01), for *A. xylosoxidans,* only piperacillin–tazobactam, meropenem and trimethoprim–sulfamethoxazole can be interpreted as being susceptible at standard dosing regimen (S), susceptible at high dosing regimen (I), or resistant (R). Among our isolates, 82.02% (260/317) were considered susceptible at standard dosing regimen to piperacillin–tazobactam. The remaining 17.98% (57/317) isolates were considered resistant to piperacillin–tazobactam. Regarding meropenem 82.75% (259/313) of our isolates were considered susceptible at standard dosing regimen, 8.63% (27/313) were considered susceptible at high dosing regimen, and another 8.63% (27/313) were considered resistant. The susceptibility to trimethoprim–sulfamethoxazole was only evaluated for 18 isolates. Among these isolates, 61.11% (11/18) were considered susceptible to trimethoprim–sulfamethoxazole. The remaining 38.89% (7/18) isolates were considered resistant to trimethoprim–sulfamethoxazole. Table 1 displays the relative and absolute amount of isolates that could be considered resistant to piperacillin–tazobactam and meropenem, grouped by specimen type. The resistance rates to both substances were the highest in stool specimens.

Among the 57 isolates that were considered resistant to piperacillin–tazobactam, 21 were still susceptible to meropenem at standard dosing regimen, and 14 were considered susceptible at a high dosing regimen. Among the 27 isolates that were considered resistant to meropenem, only five were still susceptible to piperacillin–tazobactam. Among the 22 isolates that were resistant to both substances, one was susceptible to trimethoprim–sulfamethoxazole; according to the EUCAST PK-PD breakpoint tables, nine other isolates were susceptible to a standard or high dosing regimen of at least one other tested substance. The remaining 10 isolates were not susceptible to any tested substance. The isolates were not molecularly tested to investigate the presence of resistance genes.

### 2.3. Patient Groups Detected with Achromobacter Isolates

**Figure 4:**
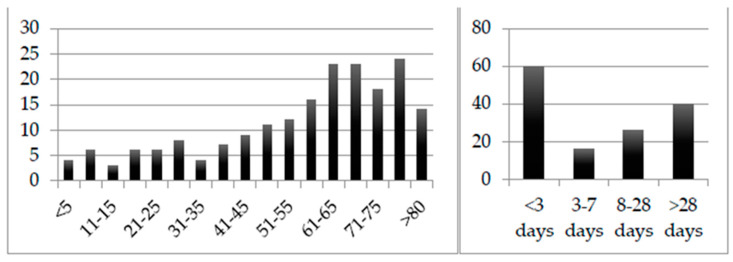
Number of isolates detected in non-screening specimens grouped by patient age (**left**) and by length of hospital stay previous to detection (**right**).

Regarding 194 of the first isolates detected in non-screening specimens, information on the patient’s age and type of clinic/ward could be retrieved. Figure 4 depicts the number of isolates detected across the different age groups. Regarding 192 of the first isolates, information on the patient’s gender could be retrieved. Of these, 112 belonged to male patients and 80 belonged to female patients. A total of 142 patients were inpatients, and 52 were outpatients. Of the outpatients, 13 were patients of the transplant outpatient clinic. From the outpatients, *Achromobacter* isolates were isolated in all but three cases from urine specimens (23), swabs (17), or stool (nine). Oncological patients made up more than one quarter (25.26%; 49/194) of the patients from which *Achromobacter* isolates were isolated, while 11.86% (23/194) of the patients were in intensive care at the time of the sample collection. Four samples were collected from oncological patients in intensive care, one of which was a blood culture. Of the remaining 64 oncological or intensive care patient samples, none was a blood culture. Of the 49 *Achromobacter* isolates detected in oncological patients, 37 (75.51%) were isolated from stool specimens.

Regarding all 142 inpatients, information on the length of hospital stay prior to the sample collection in which the *Achromobacter* isolates were isolated could be retrieved; this is displayed in Figure 4. More than one quarter of the patients (28.17%; 40/142) were detected to be colonized with *Achromobacter* isolates for the first time after more than four weeks of hospitalization; 42.25% (60/142) were assumed to have carried it on admission. Of the 40 patients that can be assumed to have been colonized by *Achromobacter* isolates after more than four weeks of hospital stay, 14 were oncological and nine were intensive care patients.

## 3. Discussion

*A. xylosoxidans* is more and more frequently referred to as an important emerging pathogen. Although most of the attention in the literature in this context has been focused on patients with cystic fibrosis [1,2,3,10,13,15,17,18,19], the microorganism has also been shown to pose an increasingly important threat to numerous other patient groups, among which—most importantly—are those with underlying oncological malignancies [7,8,9,11,14,20] and those in intensive care units [12,16,21]. Our data do not corroborate this notion, but our study cannot be compared to the majority of studies in the literature, given that cystic fibrosis patients—the most important patient group in this context—are underrepresented in our study. Furthermore, our study suffered from numerous modifications of practices during the considered period. In our setting, we attribute the frequent claim of increasing prevalence [2,13,22,23,24] to a number of changes in routine diagnostics. First, screening for Gram-negative multidrug-resistant pathogens was markedly expanded, starting in 2013. In fact, the microbiological diagnostic request to screen for these pathogens was issued for the first time in 2013, and from then on regularly, which lead to the sample material being plated on (for example ESBL) selective media (selecting with cefpodoxime). In 2013 alone, the request was placed for 11.066 samples. Microorganisms growing on the mentioned selective media were then, by default, identified with the MALDI-TOF MS that was introduced in that same year, and tested in parallel susceptibility with the Vitek 2 (both: bioMérieux, Marcy-l’Etoile, France). The introduction of the MALDI-TOF mass spectrometry in the routine identification of clinical bacterial isolates in late 2012 might also have played a role, albeit small, in the increased number of identifications, given that mass spectrometry reliably identifies a broader range of pathogens compared to biochemical identification [25,26,27,28], and the Vitek 2 system has been described as being error prone in the accurate identification of less-common opportunistic human pathogens and less-pathogenic nonfermenters, such as those belonging to the *Alcaligenaceae* [28,29,30]. Approximately one third of the *Achromobacter* isolates were detected in screening samples, which tripled from 2012 to 2013, and continued to increase over the years to nearly twice the number from 2013 in 2020. The overall amount of clinical samples likewise increased by 50% from 2012 to 2013, and another 35% from 2013 to 2020. Nevertheless, selection pressure from antibiotic therapy and the longer survival of cystic fibrosis patients [15], oncological and immunosuppressed patients could also have contributed a certain amount.

With all of its limitations, to a certain degree at least, the specimen type can hint at whether clinical suspicion of the infection existed at the time of the specimen collection, and thereby to the possible relevance of the finding. Detections of *Achromobacter* isolates in specimen types—such as tissue samples or blood cultures—that under normal conditions would be expected to be sterile, can better be assumed to be relevant findings than screening and surveillance swabs from healthy skin, for example. The collection and submission of a tracheal secretion for microbiologic analysis, for example, generally requires—at a minimum—that a patient be intubated or tracheotomized, and often does mean that a pneumonia is clinically suspected. Likewise, collecting a urine sample and requesting a microbiological analysis implies—at the very least—that a urinary tract infection (UTI) is to be ruled out, and instead in the majority of cases are linked to the patient having symptoms of a UTI or the laboratory parameters being suggestive of a UTI. Stool was the second most common specimen type that isolates were collected from. We rationalize this observation by the fact that, due to the intrinsic resistance of *Achromobacter* spp., intestinal colonization is likely to occur readily in the course of prolonged antibiotic therapies, which are common in hospitalized patients. In fact, three quarters of detections in oncological patients—who form one of the patient groups that receive the most antibiotics—were from stool. We believe that intestinal carriage might generally be underestimated. The third and fourth most-common sites of isolation were the respiratory tract and wounds; respiratory tract infections (RTIs), UTIs and wound infections also represent the three most common types of infections caused by the two most important non-fermenting nosocomial pathogens, *Acinetobacter baumannii* and *Pseudomonas aeruginosa* [31,32]. In many of the samples which were indicative of serious infections—such as those of the eye, ear, bone, central nervous system, abdomen and bloodstream—*Achromobacter* isolates were not the only detected pathogens; more importantly, the absolute number of isolates grown from such samples was minute. Furthermore, *Achromobacter* isolates representing the contamination of the sample or the only pathogens detected due to antibiotic therapies must be considered.

The phenotypic antibiotic susceptibility values revealed in our study coincide remarkably well with genotypic data from the literature. *Achromobacter* species chromosomally encode two well-characterized multidrug efflux pumps [33]. Both are found on all of the publicly available *Achromobacter* genomes, even in fully susceptible strains [34,35]. AxyXY-OprZ is a broad-spectrum efflux pump which is most importantly responsible for resistance against aminoglycosides. It shares a high similarity with the MexXY/OprM efflux pump from *P. aeruginosa* but appears to confer a much higher level of aminoglycoside resistance. In our study, a few isolates had very low MIC values to aminoglycosides; hence, these could have been misidentified as *Achromobacter* isolates. Apart from aminoglycosides, the AxyXY-OprZ broad-spectrum efflux pump also confers decreased susceptibility to carbapenems, cefepime, some fluoroquinolones, tetracyclines, erythromycin, and—to a lesser extent—ceftazidime [36]. The AxyABM efflux pump instead plays a major role in the extrusion of cephalosporins other than cefepime and cefuroxime, and in the extrusion of aztreonam [35]. Our study lacks a molecular analysis of isolates, but phenotypically the vast majority of *Achromobacter* isolates, indeed, had MIC values above the maximum measurable value for amikacin, gentamicin and tobramycin (see Figure 3b).

A deletion in an upstream transcriptional regulator gene, *axyZ,* causing an amino acid substitution has been shown to lead to AxyXY-OprZ overproduction, which is associated with increased MICs to fluoroquinolones, cefepime, and tetracyclines [37]. The MIC values of ciprofloxacin, levofloxacin and moxifloxacin in our study were largely above 2µg/mL (see Figure 3b,c). The ceftazidime, cefepime, ampicillin–sulbactam, tigecycline and Imipenem MIC values show bimodal distributions and a single amino acid substitution leading to an up to 17-fold transcription level increase of the AxyXY-OprZ efflux pump [37], could certainly explain these findings.

Furthermore, OXA-114-like β-lactamases are chromosomally encoded on *Achromobacter* genomes. Their main substrates in vitro are generally penicillin, narrow-spectrum cephalosporins, piperacillin, and even imipenem, although to a much lesser degree. This chromosomally encoded oxacillinase nevertheless does not seem to phenotypically render *A. xylosoxidans* resistant to these substances by itself [35,38,39]. In our study, the isolates mostly had ampicillin MICs ≥8µg/mL, as well as very high MICs for first, second and third generation cephalosporins, but piperacillin and piperacillin–tazobactam MICs ≤4 µg/mL. Comparing the MIC distributions, the β-lactamase inhibitor seemed to lower the MICs of the ureidopenicillin only very slightly, if at all, with one limitation being that the two substances were not directly compared in the study.

In addition, *A. xylosoxidans* has been described as being capable of acquiring important β-lactamases, such as ESBLs, cephalosporinases and carbapenemases (especially metallo-β-lactamases) [39,40,41,42,43,44,45,46,47,48,49]. In our study, the isolates showed meropenem MICs largely below the lowest measurable concentration of the VITEK2 cards of 0.25µg/mL but imipenem MICs most commonly at 1µg/mL or 8µg/mL (see Figure 3b). The ertapenem MIC distribution resembled more closely that of meropenem compared to imipenem. Aztreonam, the only β-lactam antibiotic that is generally not inhibited by metallo-β-lactamases, had in all but a few cases MICs ≥32µg/mL, being a target of the chromosomally encoded AxyABM efflux pump.

According to the EUCAST clinical breakpoints v11.0 (01.01.2021), only the MICs of piperacillin–tazobactam, meropenem and trimethoprim–sulfamethoxazole may be interpreted for *A. xylosoxidans*. We only have 18 MIC determinations for trimethoprim–sulfamethoxazole, as opposed to 317 for piperacillin–tazobactam and 313 for meropenem. Besides the publicly available EUCAST MIC distribution data, we only found two previous studies providing information on the susceptibility to piperacillin–tazobactam and meropenem for at least 30 isolates. One summarized susceptibilities declared in previous studies on *A. xylosoxidans* bacteremia without reporting MIC values. It stated a 94% susceptibility for piperacillin–tazobactam and meropenem, and a 9% susceptibility for trimethoprim–sulfamethoxazole [14]. The other covered 63 non-respiratory clinical isolates, and reported 100% susceptibility to piperacillin–tazobactam and 97% susceptibility to meropenem [50]. In our study, the isolate susceptibility for piperacillin–tazobactam—and even moreso, meropenem—strongly fluctuated depending on the specimen that the isolate was derived from. The isolates detected in stool specimens displayed the largest amount of resistances, which was certainly biased by the fact that the routine culture media on which stool samples from oncological patients are plated already select for more-resistant organisms. The resistance rates detected in isolates from stool are, hence, not representative.

Twelve isolates were not susceptible to piperacillin–tazobactam, meropenem, trimethoprim–sulfamethoxazole, or any other tested substance (according to EUCAST Clinical Breakpoint Tables v. 11.0 for *A. xylosoxidans* and PK-PD, respectively). The acquisition of one or more mobile genetic elements could explain such resistance patterns. The lack of a molecular analysis of these isolates represents a limitation, and highlights the need for more frequent molecular analysis, and of species other than those which frequently carry carbapenemase-encoding plasmids.

As far as colonization/ infection patterns are concerned, two main patterns can be distinguished in the literature for *A. xylosoxidans*. On the one hand, patients generally seem to carry their own individual strain, depending on the type of colonization, even for several years. Cystic fibrosis patients, for example, are assumed to acquire one of several environmental strains early in life, and maintain that particular strain [17]. Two patients that underwent lung transplantation have even been described to have been recolonized with their individual pre-transplant strain after transplantation [13]. Co-colonisation with different strains is only sporadic, and is limited to shorter periods [17]. Other *Achromobacter* species seem to be less capable of chronically colonizing patients [19]. On the other hand, *A. xylosoxidans* can be acquired in the course of nosocomial outbreaks [3,14,20,21,51]. In these cases, patients are infected by one of a few clonally related isolates that have spread globally throughout several healthcare facilities, such as the Belgian epidemic clone AxST137 that has also spread to France [18]. Some clonal strains even seem capable of causing deterioration in lung function [1,52]. In our study, the amount of *A. xylosoxidans* that were carried on admission approximately equaled those that were detected after more than a week of hospitalization, which does not quite paint the pattern of a nosocomial pathogen. The isolates were not molecularly analyzed for their strain or clonality; however, future studies should focus on the examination of whether nosocomially acquired isolates are clonally related to each other, or even to other clonal strains found throughout Europe. As for cases in which *A. xylosoxidans* is detected after prolonged hospital stays, oncological patients would appear to be the most severely affected patient group globally [7,8,9,11,14,20,21]. Because these patients are more vulnerable to opportunistic infections, they receive antibiotic courses more frequently than other patient groups. These eradicate most potential opportunistic pathogens but also damage the host’s beneficial and protective microflora, and can ultimately lead to environmental bacteria with intrinsic resistance mechanisms, such as *A. xylosoxidans* to be capable of settling down in the gastrointestinal tract [53,54,55,56,57,58]. These patient groups will therefore benefit from further studies on the hospital microenvironment in order to identify and seal potential sources of infection, and from studies on how protective microbiome factors can be maintained despite antibiotic therapies.

The limitations of the study include the fact that it is a single-center study that retrospectively compared minimum inhibitory concentrations of isolates tested at their respective times according to the specifications and methods of the time. The MIC determination for routine diagnostics was routinely performed with the VITEK 2 system, and the susceptibility testing systems were commercially available test systems and not in-house systems, the accuracy of which, however, should be comparable to the latter. Most of the piperacillin–tazobactam MIC values were Vitek2 results, which are known to be of limited quality; during the study period, the use of the Vitek2 to determine the piperacillin–tazobactam susceptibilities was prohibited by the FDA for about a year; likewise, a stern warning on the limitations for gradient strip tests has been active for a couple of years. The piperacillin MIC results, however, would seem to indicate that, in general, the results of the piperacillin–tazobactam tests can be quite orientating. The isolates were not confirmed as *A. xylosoxidans* by MLST-typing or nrdA gene sequencing; they were only identified biochemically until 2013, and by MALDI-TOF MS from 2013 onwards.

## 4. Materials and Methods

### 4.1. Data Retrieval

We analyzed retrospective data on *Achromobacter xylosoxidans* isolates that could be retrieved from the laboratory information system of our institute, which is part of the University Hospital of Bonn, Germany (UKB). The UKB is a tertiary referral and maximum care hospital with 1300 beds. Every year, about 50,000 inpatients and 35,000 emergencies are treated, and over 350,000 outpatient treatments are provided. Our microbiological diagnostic unit services the University Hospital Bonn and other hospitals in the area; for example, it received 203,992 microbiological samples in 2020. All of the first isolates from 1 January 2004 to 6 December 2021 were retrieved from the laboratory information system using the current and past (*Alcaligenes xylosoxidans*) species name, by one operator. In parallel, for each isolate, information on the minimum inhibitory concentrations (MIC) for all tested antibiotics, information on the number of bacterial or fungal species detected in the respective specimen beside the *Achromobacter* isolate, patient age, type of clinical specimen, and requested microbiological diagnostics were retrieved. For each patient, only the first detected isolate was considered.

### 4.2. Ward Type and Length of Hospitalization

The isolate information for non-screening-specimens was complemented with information on whether the submitting clinic was oncological, an intensive care unit, or the transplant outpatient clinic; information on the in- or outpatient status; the gender of the patient the sample was collected from; and the length of hospital stay prior to the sample collection in which the *Achromobacter* isolates were isolated. The generated dataset complied with all of the basic ethical guidelines and expectations, and did not disclose any patient-identifying information. The information contained in the dataset was confined to the year of detection, the sample type, the age in years, and the sex of the patient; whether the specimen was submitted by an oncology department, an intensive care unit, or the transplant outpatient clinic; the length of hospital stay prior to the sample collection in which *Achromobacter* isolates were first isolated; and the minimum inhibitory concentrations of the tested compounds, if applicable.

### 4.3. Minimum Inhibitory Concentrations of the Antibiotics

Before 2013, the susceptibility of *A. xylosoxidans* to antimicrobial compounds was routinely measured by disk diffusion, which limits the data on MICs in the period of 2004 to 2012 to those exceptional cases in which testing was performed using gradient-strip tests; the isolate identification was performed biochemically. Starting from late 2012, diagnostic microbiological samples were routinely cultured on standard laboratory growth media, and isolates were identified via MALDI-TOF MS (VITEK MS, bioMérieux, Marcy-l’Etoile, France) and susceptibility tested with the VITEK 2 system (bioMérieux, Marcy-l’Etoile, France). Inconsistent results of routine susceptibility analyses were routinely replaced or supplemented with gradient strip tests. MIC Test Strips from Liofilchem were used (except for piperacillin–tazobactam (bioMérieux)), and were carried out on Mueller–Hinton agar plates (Becton Dickinson).

The ethics committee of the University Hospital Bonn confirmed that no ethics approval was required for this study.

All data relevant to the study are included in the article.

## 5. Conclusions

We retrospectively analyzed data on clinical *Achromobacter* spp., labeled as *A. xylosoxidans* by MALDI-TOF MS and/or biochemically, detected during an 18 year period in our diagnostic unit, along with associated demographic and clinical information and the results of routine MIC determination. We found that the apparent increase in prevalence, in our setting—in which cystic fibrosis patients were underrepresented—may in large part simply be attributable to an overall increase in the number of samples and, more importantly, the introduction of rigorous screening for Gram-negative multidrug-resistant pathogens. We found the resistance rates to piperacillin–tazobactam and meropenem to vary strongly between different sample types and a surprisingly high number of isolates in stool, hinting at the possibility that intestinal carriage might generally be underestimated. Isolates that are resistant to meropenem can hardly be treated, and should be analyzed molecularly in order to detect and prevent the spread of mobile resistance genes or high-risk clones.

## Figures and Tables

**Table 1:** Relative and absolute amounts of the isolates which were considered resistant to piperacillin–tazobactam and meropenem according to EUCAST Cl. Br. Tables v. 11.0, grouped by the specimen type.

Specimen Type	Amount of Isolates Resistant to	
	Piperacillin–tazobactam	Meropenem
Blood	1/7	0/7
Urine	15.69% (8/51)	4.08% (2/49)
Stool	37.21% (16/43)	16.28% (7/43)
Screening	17.09% (20/117)	7.76% (9/116)
All specimens	17.98% (57/317)	8.63% (27/313)

## Data Availability

All data relevant to the study are included in the article.
